# Medical Association of Georgia

**Published:** 1875-11

**Authors:** 


Medical Association of Georgia.—The minutes of the twenty-sixth annual meeting of the Medical Association of Georgia have been received. It will be seen from the following extract of a note by the Publishing Committee, that the financial condition of the Association is such as to preclude the publication of the valuable papers presented at the meeting in Savannah last April:

"The Committee on Publication regret that lack of funds precludes the publication of the Transactions in full. The minutes have been withheld till now with the hope that the required funds might be obtained. The material for an interesting volume is abundant and excellent—more so, perhaps, than ever before in the history of the Association."

This is to be regretted; but nothing more could have been expected after the statements of the Secretary and Treasurer, when the subject of assessments was under consideration at the last meeting. As one of the officers of the Association in 1874, I have a feeling recollection of the difficulties encountered in issuing the Transactions, which were, after a delay of several months, published in part, with the private funds of the officers. Out of the five or six hundred names, which we from year to year find on our roll of members, it does appear strange that dues sufficient cannot be collected to publish the Transactions; but such is the case, I am sorry to know. The only way to remedy this defect, and save the Association, is to increase the assessments, as under the present a sufficient amount can not be collected. From the note above referred to by the Publishing Committee, we make the following extract, which will remedy the whole trouble, and place the Association on an enduring basis, if Dr. Ford is successful in changing the By-Laws:

"The President, Dr. Ford, requests the committee to announce that at the next annual meeting he will offer to amend the By-Laws, so that the initiation fee shall be five dollars and the annual assessment five dollars; and that failure to comply for two years shall forfeit membership."

